# Bi Nanoparticles Anchored in N-Doped Porous Carbon as Anode of High Energy Density Lithium Ion Battery

**DOI:** 10.1007/s40820-018-0209-1

**Published:** 2018-06-08

**Authors:** Yaotang Zhong, Bin Li, Shumin Li, Shuyuan Xu, Zhenghui Pan, Qiming Huang, Lidan Xing, Chunsheng Wang, Weishan Li

**Affiliations:** 1 School of Chemistry and Environment, South China Normal University, Guangzhou, 510006 People’s Republic of China; 2 Engineering Research Center of MTEES (Ministry of Education), Research Center of BMET (Guangdong Province), Engineering Laboratory of OFMHEB (Guangdong Province), Key Laboratory of ETESPG (GHEI), and Innovative Platform for ITBMD (Guangzhou Municipality), South China Normal University, Guangzhou, 510006 People’s Republic of China; 3 Department of Chemical and Bimolecular Engineering, University of Maryland, College Park, College Park, MD 20740 USA

**Keywords:** Porous N-doped carbon, Bi nanoparticles, Anode, Lithium-ion battery, High energy density

## Abstract

A novel bismuth–carbon composite, in which bismuth nanoparticles were anchored in a nitrogen-doped carbon matrix (Bi@NC), is proposed as anode for high volumetric energy density lithium ion batteries (LIBs). Bi@NC composite was synthesized via carbonization of Zn-containing zeolitic imidazolate (ZIF-8) and replacement of Zn with Bi, resulting in the N-doped carbon that was hierarchically porous and anchored with Bi nanoparticles. The matrix provides a highly electronic conductive network that facilitates the lithiation/delithiation of Bi. Additionally, it restrains aggregation of Bi nanoparticles and serves as a buffer layer to alleviate the mechanical strain of Bi nanoparticles upon Li insertion/extraction. With these contributions, Bi@NC exhibits excellent cycling stability and rate capacity compared to bare Bi nanoparticles or their simple composites with carbon. This study provides a new approach for fabricating high volumetric energy density LIBs.

**Electronic supplementary material:**

The online version of this article (10.1007/s40820-018-0209-1) contains supplementary material, which is available to authorized users.

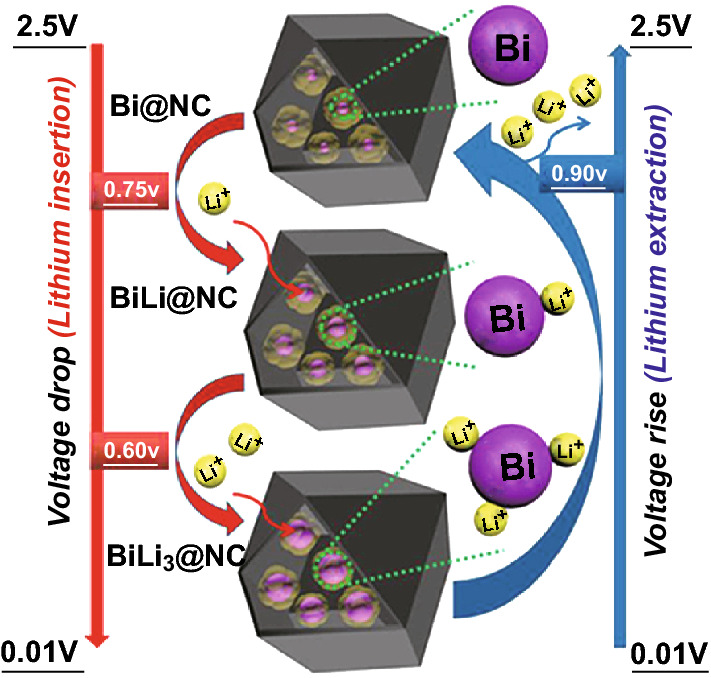

## Highlights

- The Bi nanoparticles anchored in N-doped porous carbon (Bi@NC) composite was prepared by a facile replacement reaction method, in which ultrasmall Bi nanoparticles were homogeneously encapsulated in the carbon matrix
- The N-doped carbon matrix enhanced the electric conductivity and alleviated the mechanical strain of Bi nanoparticles on Li insertion/extraction due to the larger void space, and Bi@NC exhibits excellent cyclic stability and rate capability for LIBs
- The strategy developed in this work solves the cyclic instability issue of bismuth as anode for LIBs and provides a new approach to improve high volumetric energy density for electrochemical energy storage devices.

## Introduction

Power sources with high volumetric and gravimetric energy densities are urgently needed to meet the small size and long service life requirements of various applications from information technology to transportation [1–6]. Lithium-ion batteries (LIBs) are the dominant power sources for these applications owing to their superior energy densities and cycle lives compared to other secondary batteries, but their energy densities are still unsatisfactory for quickly developing society [7–11].

Graphite is the most commonly used anode in commercial LIBs because of its superior cycling stability and high coulombic efficiency. However, the low theoretical capacity of the graphitic anode (372 mAh g^−1^) limits the development of graphite-based LIBs. Therefore, it is necessary to look for high energy density LIBs anodes.

**Fig. 1:**
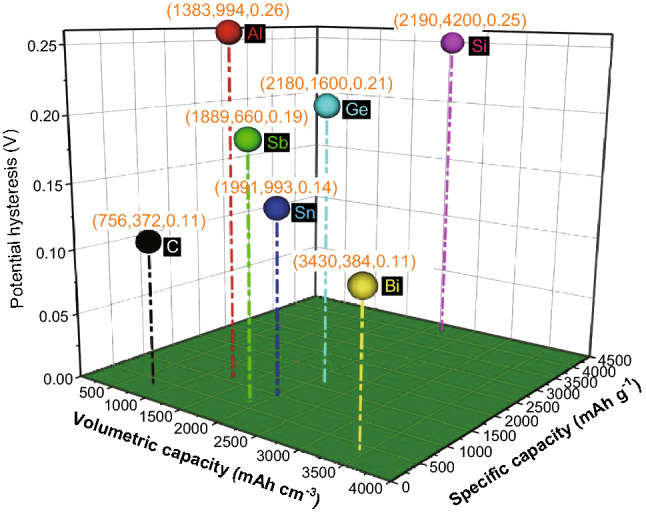
Lithium storage performances of various metals in comparison to graphite

Several metals including Al, Si, Sn, Sb, Ge, and Bi have captured attention as anode materials due to their high theoretical capacities compared to graphite, which has been used as anode since the invention of LIBs. Al, Si, Sn, Sb, and Ge have far higher theoretical gravimetrical capacities than that of graphite (372 mAh g^−1^) through the formation of LiAl (994 mAh g^−1^), SiLi_4.4_ (4200 mAh g^−1^), SnLi_4.4_ (993 mAh g^−1^), SbLi_3_ (660 mAh g^−1^), and Li_2.2_Ge_5_ (1600 mAh g^−1^), but cannot give correspondingly high volumetric capacities, which is only respective 1383, 2190, 1991, 1889, and 2180 mAh cm^−3^ compared to 756 mAh cm^−3^ of graphite [12, 13]. Besides, these metals yield potential hysteresis of 0.26, 0.25, 0.14, 0.19, and 0.21 V for lithiation/delithiation, respectively, which are not only larger than in graphite (0.11 V), but also are energy inefficient [1]. Although Bi is a diagonal element of Sn and in the same group as Sb, it has unique layered crystal structure that can provide larger interlayer spacing to accommodate Li ions (such as Li_3_Bi) [14–16]. Most importantly, bismuth gives a volumetric capacity of 3430 mAh cm^−3^, which is far higher than those of other metal anodes and about five-bold than that of graphite [17]. It also yields potential hysteresis the same as graphite [18] although its specific capacity (385 mAh g^−1^) is not so high, as shown in Fig. 1. These features of bismuth make LIBs attractive in applications where high volumetric energy densities are required [19–21].

Like other metal anodes, however, bismuth exhibits poor cycling stability due to its large volume change during lithiation/delithiation [1]. Some efforts have been made to solve this problem. For example, Park et al. [21] prepared a nanostructured Bi@C composite that delivered a relatively high capacity of 300 mAh g^−1^ after 100 cycles at current density 100 mA g^−1^ by varying the voltage from 0.0 to 2.0 V. Yang et al. [22] revealed that Bi@C microspheres as anode materials for LIBs retained capacity of 280 mAh g^−1^ after 100 cycles at current density 100 mA g^−1^. The improved cycling stability of bismuth in these efforts can be attributed to the controlled coating of carbon layer on bismuth, which enhances electronic conductivity and alleviates the mechanical strain of bismuth during lithiation/delithiation [23, 24]. Moreover, the controlled coating of carbon layer acts as host to stabilize the solid electrolyte interphase (SEI) on the bismuth surface [25]. However, the above-mentioned achievements are unsatisfactory for the practical application of bismuth as anode in LIBs.

Various carbon materials have been extensively studied for performance improvement of anode or cathode materials in LIBs [26–30]. Metal organic frameworks (MOFs) characterized by diverse skeletal structures, high surface areas, tunable pore sizes, and open metal sites in the skeleton have been demonstrated as promising templates or precursors for fabricating nanostructured carbon for various applications [31–37]. Except for the advantages mentioned above, MOFs can also be designed and synthesized in a straightforward and cost-effective manner by assembling varied metal ions/clusters and organic ligands under mild conditions [38]. Therefore, without any processing equipment, it can be simply mass-produced just by increasing the amounts of raw materials. In addition, it has been noted that nitrogen-containing MOFs yield nitrogen-doped carbon that exhibits enhanced electronic conductivity and activity toward reactions on carbon [39, 40]. Zeolitic imidazolate framework (ZIF-8), a kind of nitrogen-containing MOFs, combines high stability of inorganic zeolite with high surface area and porosity, and is a good precursor for preparing carbon matrices to enhance cycling stability of some electrode materials for LIBs [41–43]. For example, Si@ZIF8 composites were prepared by Han et al. [44] via in situ mechanochemical synthesis, which shows superior electrochemical properties with lithium storage capacity up to 1050 mAh g^−1^ and excellent cycle stability (> 99% capacity retention after 500 cycles).

In this work, a novel carbon/bismuth composite is introduced through a novel synthetic strategy wherein ZIF-8 was used as precursor for N-doped porous carbon to improve the cycling stability of the bismuth anode. ZIF-8 was obtained by a simple hydrothermal method at low temperature and underwent pyrolysis in H_2_/Ar atmosphere to form N-doped porous carbon with dispersed zinc nanoparticles. Based on the potential difference between redox couples of Zn^2+^/Zn (− 0.76 V vs. SHE) and Bi^3+^/Bi (0.31 V) [45], bismuth nanoparticles were anchored on the carbon matrix through a replacement reaction. The carbon matrix afforded an electronically conductive network and served as support to restrain the aggregation of bismuth nanoparticles [46]. Most importantly, the pores in the carbon matrix provided space to alleviate the mechanical strain of bismuth during lithiation/delithiation. With these features, the resultant carbon/bismuth composite exhibited excellent performance as anode for LIBs when compared to other bismuth anodes that have been reported in other literatures.

## Experimental Section

### Sample Syntheses

ZIF-8 was synthesized hydrothermally [26]. Typically, 3 mmol zinc nitrate hexahydrate (Zn(NO_3_)_2_·6H_2_O, 99%) and 8 mmol 2-methylimidazole (MeIm, 98%) were separately dispersed in 40 mL methanol (99.5%) with moderate magnetic stirring for 10 min and then mixed under stirring for another 30 min at room temperature. The mixture was sealed in a Teflon−lined autoclave and maintained at 100 °C. After a certain period of time, a white precipitate was harvested by centrifugation at 8000 rpm for 3 min, thoroughly washed with methanol, followed by drying in a vacuum oven overnight.

To obtain N-doped porous carbon with dispersed zinc nanoparticles (Zn@NC), carbonization process was carried out. The as-obtained ZIF-8 was heated at 500, 600, 700, and 800 °C for 3 h at the rate 2 °C min^−1^ under H_2_/Ar atmosphere with slow flow. Finally, a tan product was produced after high temperature calcination.

Bismuth nanoparticles were anchored in N-doped porous carbon matrices by galvanic replacement reaction. Typically, 1 mmol as-obtained Zn@NC and 1 mmol BiCl_3_ were homogeneously dispersed in 75 mL mixed solvent of glycerin and methanol (2:1 in volume) under ultrasonic treatment at room temperature for 30 min. The mixture was sealed in a 100 mL Teflon−lined autoclave, maintained at 120 °C for a certain period of time and then cooled naturally. To obtain the product (Bi@NC), the precipitation was thoroughly washed with methanol via centrifugation–redispersion cycles at 9000 rpm for 5 min and finally dried in a vacuum oven overnight.

The NC sample was obtained by washing Zn@NC with dilute HCl and then deionized water several times to remove the residual Zn component.

For performance comparison, Bi nanospheres (bare Bi, Beijing Dekedao, 99.95%, OD 100 nm) were used and a bismuth/carbon composite (Bi@C) was prepared hydrothermally by coating Bi nanospheres with carbon. Typically, 0.63 g Bi nanospheres were dispersed in 15 mL deionized water, which was mixed with 48 mL aqueous solution containing 1.8 g glucose. Methanol (15 mL) was added under stirring at room temperature for 15 min. The mixture was then sealed in a 100 mL Teflon-lined autoclave and heated at 190 °C for 15 h. After cooling naturally, the precipitate harvested as Bi@NC was prepared. Finally, the product Bi@C was obtained by heating the precipitate at 550 °C for 3 h under N_2_ at the rate 2 °C min^−1^.

### Physical Characterizations and Electrochemical Measurements

The crystal configurations and crystallographic planes of the synthetic materials were identified by X-ray diffractometry (XRD, Ultima IV Germany). The specific surface area and pore diameter distribution were tested at liquid nitrogen temperature (77 K) with a surface area and porosimetry analyzer (V-Sorb 2800P). Scanning electron microscopy (SEM, JEOL JSM-6380LA) and transmission electron microscopy (TEM, JEOL JEM-2100HR) were carried out to observe the morphologies, structures, and particle sizes of the samples. During SEM observation, energy dispersion spectrum (EDS) and EDS mapping were also obtained. Fourier transition infrared (FTIR) spectrum of ZIF-8 was determined using infrared spectroscopy (Bruker Tensor 27) within 500–4000 cm^−1^. X-ray photoelectron spectrometer (XPS, Thermo Fisher Scientific, UK) was used with monochromatic Al-Kα X-ray source (excitation energy = 1468.6 eV) under ultra-high vacuum (lower than 5 × 10^−8^ mbar). Spectra were collected from 0 to 1350 eV using an X-ray spot size of 400 μm with pass energy 100 eV for wide scan and 30 eV for individual elements. Binding energies were corrected based on the carbon 1*s* signal at 284.8 eV. Raman spectra were examined on an Alpha 300R Raman instrument at room temperature. The apparent densities of the samples were obtained by keeping the samples in a volumetric cylinder and then vibrating the cylinder until the volumes of the samples remained unchanged.

The Bi electrodes were composed of active materials, bare Bi, Bi@C or Bi@NC, acetylene black, and PVDF in the ratio 7:1.5:1.5 by mass, which were mixed in *N*-methyl pyrrolidone and coated on Cu foil (*S* = 1.13 cm^2^) with the weight of active materials being about 0.5 mg. CR2025 type coin cells were assembled with Bi electrode, lithium foil electrode, electrolyte of 1.0 M LiPF_6_ in ethyl methyl carbonate (EMC)/ethylene carbonate (EC)/diethyl carbonate (DEC) (EMC/EC/DEC = 5:3:2, by weight), and a microporous membrane (Celgard 2400), in an Ar-filled glove box (Vigor-CH) where water and oxygen contents were controlled to less than 0.1 ppm.

The assembled coin cells were patiently tested on a multi-channel battery tester (LAND CT2001A, Wuhan, China) at 25 °C by discharging to 0.01 V and charging to 2.5 V at various current rates. Under certain operation conditions, cyclic voltammetry (CV) was collected from multichannel potentiostats (Bio-Logic SAS VMP-3) at scan rate 0.1 mV s^−1^. The electrochemical impedance spectroscopy of coin cells was carried out on an Autolab (PGSTAT302N) with AC signal 10 mV_rms_ from 0.1 MHz to 0.01 Hz.

## Results and Discussion

**Fig. 2:**
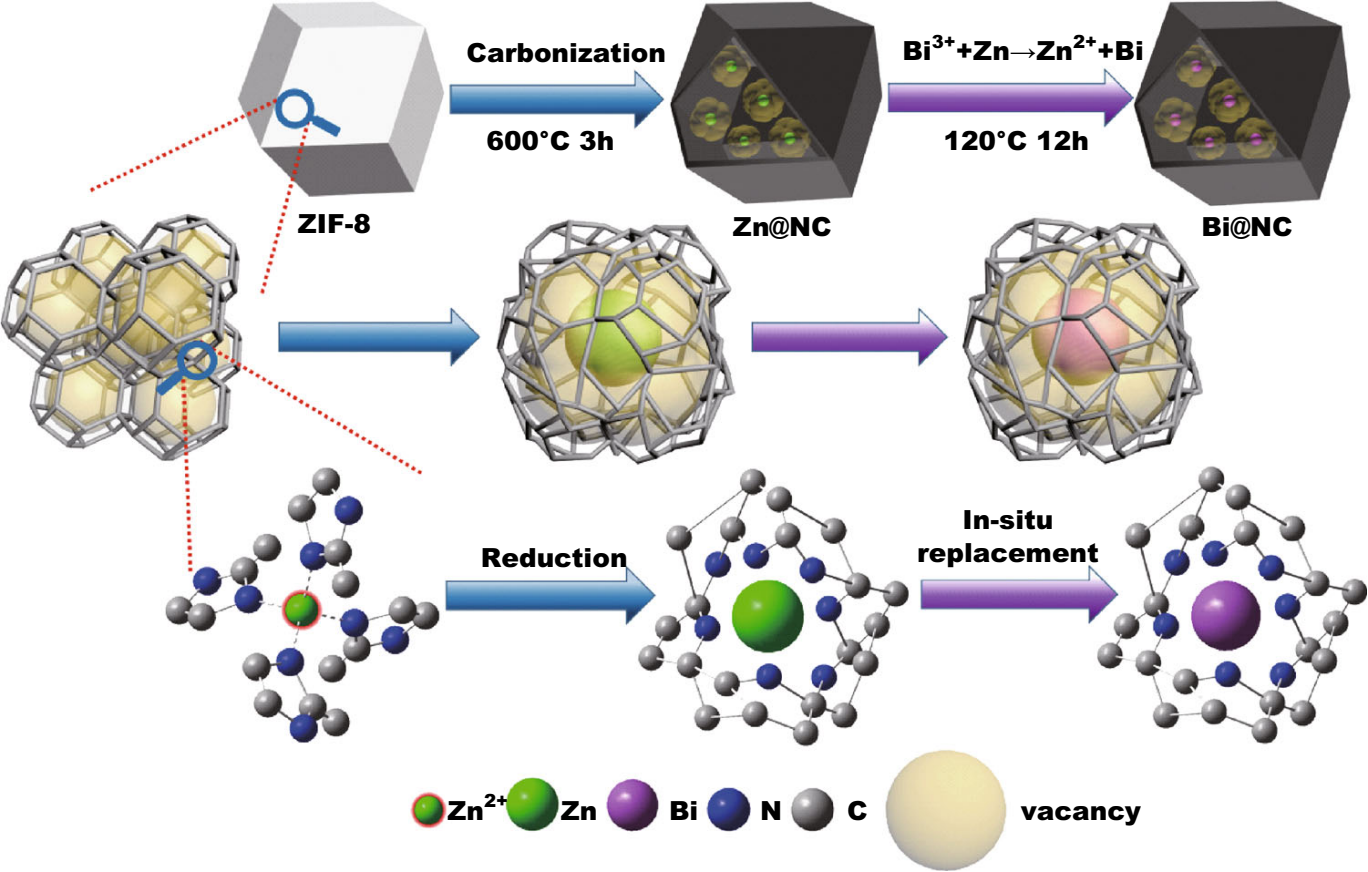
Schematic illustration of the formation process of Bi@NC

The synthetic route for Bi@NC is depicted in Fig. 2. ZIF-8 was used as precursor and Zn@NC was obtained via carbonization of ZIF-8 under H_2_/Ar. The polyhedral morphology of ZIF was maintained and the skeleton was composed of nitrogen-doped carbon. The Zn^2+^ ions in the ZIF-8 precursor were transformed to Zn nanoparticles under the effect of pyrolytic carbon as reducing agent. A galvanic replacement reaction took place, when Bi^3+^ ions were introduced. This enabled the Bi nanoparticles to replace Zn nanoparticles in situ, resulting in a special configuration of Bi nanoparticles anchored in the skeleton of nitrogen-doped porous carbon. This configuration provided the resulting Bi@NC with advantages of highly active Bi nanoparticles, electronically conductive NC, and Li insertion/extraction volume buffering porous structure.

**Fig. 3:**
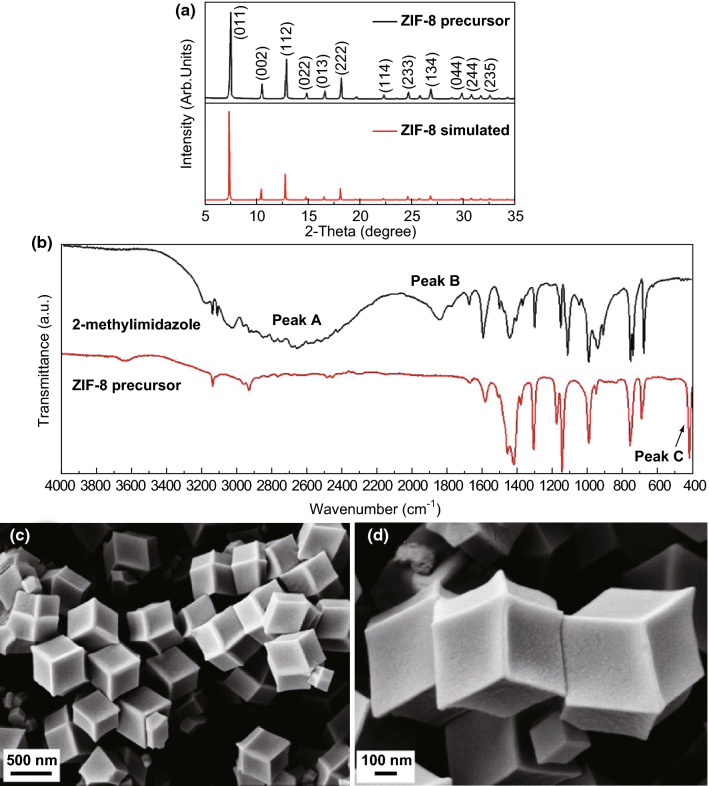
**a** XRD pattern, **b** FTIR spectrum, and **c**, **d** SEM images of ZIF-8 precursors

The synthesized ZIF-8 was characterized with XRD, FTIR, and SEM. Figure 3a presents the XRD pattern of ZIF-8 precursor, compared with simulated ZIF-8 [47–49]. It can be clearly seen from Fig. 3a that all diffraction peak intensities and shapes of synthesized ZIF-8 are identical to the simulated ZIF-8, indicating high crystallinity and purity of the ZIF-8 precursor. The intensity of peak at 7.3° referring to the (0 1 1) plane of ZIF-8 is much stronger than other peaks, illustrating an advantageous (0 1 1) plane [50]. Figure 3b presents the FTIR spectrum of the synthesized ZIF-8 with a comparison of its reactant. The synthesized ZIF-8 exhibits a different FTIR spectrum from MeIm. The wide absorption peak (Peak A) in MeIm caused by vibrations of the hydrogen bonds established between the pyrrole group and the pyridinic nitrogen (N–H…N) in the range 2200–3200 cm^−1^ completely disappeared in the synthesized ZIF-8, suggesting that Zn^2+^ successfully coordinated with MeIm [51]. Obviously, the absorption peak at about 1845 cm^−1^ (Peak B) in MeIm caused by resonance between the N–H…N bending ‘‘out of plane’’ and N–H stretching vibrations was not detected in the synthesized ZIF-8 [50, 52]. Meanwhile, a new absorbance peak at about 423 cm^−1^ (Peak C) appearing in the synthesized ZIF-8 is ascribed to Zn–N stretching. These differences further verified the bond connectivity between MeIm and Zn^2+^, as previously reported in ZIF-8 [49, 53]. As shown in Fig. 2, due to Zn *sp*^3^ hybridization, ZIF-8 exhibited a sodalite zeolite structure formed by four- and six-member ring ZnN_4_ clusters with large internal vacancies (1.16 nm in diameter) [48, 54, 55]. Apparently, the ZIF-8 crystal structure was well formed in the synthesized ZIF-8. Figure 3c presents the SEM image of the synthesized ZIF-8, showing that its particle size is uniform, about 500 nm with dodecahedral morphology. As depicted in Fig. 3d, the enlarged SEM image of ZIF-8 precursor visually displays its smooth surface, striking angular morphology, and well-defined facets.

**Fig. 4:**
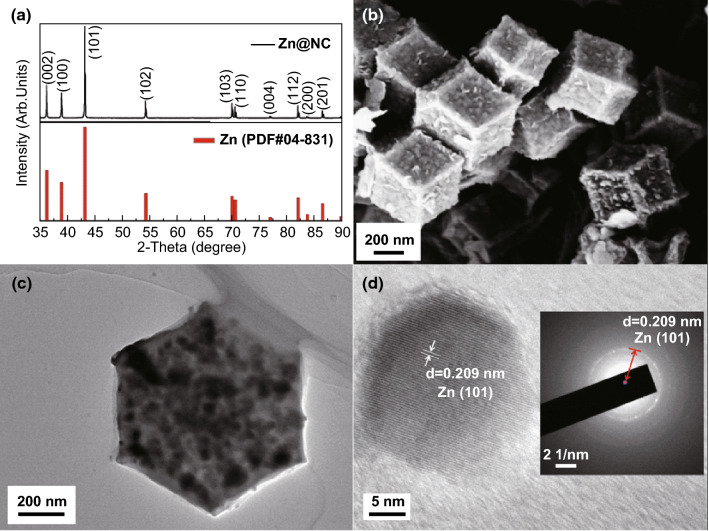
**a** XRD pattern, **b** SEM, **c** TEM, and **d** HRTEM and SAED images of Zn@NC

As shown in Fig. 2, Zn@NC was obtained by calcining the synthesized ZIF-8. From Fig. S1a to d, the Zn@NC maintains a more complete structure after ZIF-8 calcination at 600 °C, compared to those at 700 and 800 °C. In addition, although the morphology after calcination at 500 °C was the best in all samples, its degree of graphitization was lower than that of 600 °C [56]. Therefore, 600 °C was the optimum temperature for calcination. The resulting Zn@NC was characterized with XRD, SEM, and TEM, and the results obtained are shown clearly in Fig. 4. From the XRD pattern (Fig. 4a), all diffraction peak intensities and positions of Zn@NC matched with those of metallic Zn (PDF#04-0831). This identification suggested that the organic compositions of ZIF-8 were converted to pyrolytically amorphous carbon composite while the zinc ions in ZIF-8 were reduced by pyrolytic carbon to metallic zinc [57]. From the SEM image (Fig. 4b), it was observed that after the carbonization process, Zn@NC retained the pristine rhombic dodecahedron morphology of ZIF-8 but its surface became rough. The TEM image of Zn@NC (Fig. 4c) reveals that metallic zinc existed in the form of nanoparticles that are distributed in the carbon matrix. The high-resolution TEM (HRTEM) and electron diffraction images (Fig. 4d) indicate that the Zn nanoparticle was about 15 nm with *d*-spacing 0.209 nm, which corresponds to the (101) plane of Zn. Raman spectroscopy was carried out to confirm the existence and structure of carbon in Zn@NC. As shown in Fig. S2a, two scattering bands are located at 1328 and 1575 cm^−1^, which could be defined as the D and G bands of carbon, respectively. Moreover, the intensity ratio *I*_D_/*I*_G_ was estimated to be about 1.13, revealing a comparatively low degree of graphitization. This may be due to the generation of gas and re-formation of carbon structure during the carbonization process [58].

**Fig. 5:**
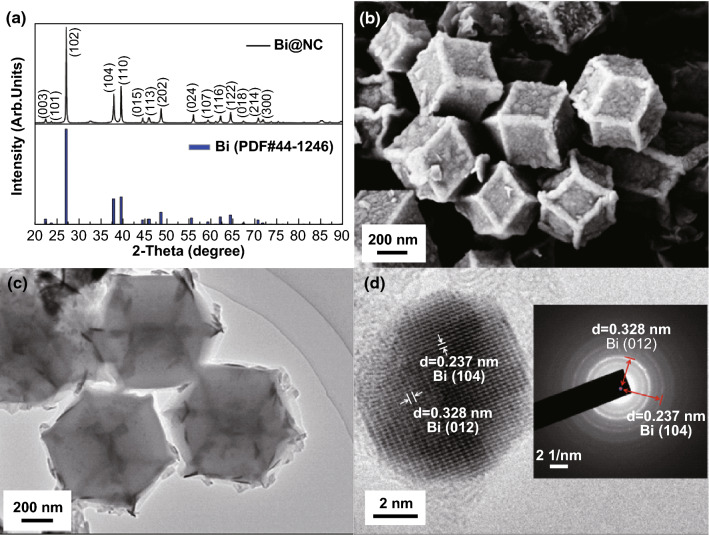
**a** XRD pattern, **b** SEM, **c** TEM, HRTEM, and **d** SAED images of Bi@NC

Bi@NC was designed by the replacement of zinc by bismuth, as indicated in Fig. 2, which was based on different potentials for bismuth and zinc. The standard hydrogen electrode potential of bismuth, E^Θ^(Bi^3+^/Bi), is 0.31 V compared to − 0.7628 V for zinc, E^Θ^(Zn^2+^/Zn). The resulting Bi@NC was characterized by XRD, SEM, TEM, EDS, XPS, and BET, which are presented in Figs. 5 and S3. The XRD pattern (Fig. 5a) illustrates that the diffraction peaks of Bi@NC match those of metallic Bi (PDF#44-1246), indicating successful in situ replacement between Bi^3+^ and Zn. The SEM (Fig. 5b) and TEM images (Fig. 5c) display that the as-prepared Bi@NC retained the rhombic dodecahedral morphology inherited from its precursor and that the Bi particles are well dispersed in Bi@NC. This configuration provided Bi@NC with an apparent density of 1.51 g cm^−3^, which is nearly twice that of graphite, whose apparent density is about 0.74 g cm^−3^. As shown in Fig. 5d (HRTEM and SAED images), the Bi nanoparticles in Bi@NC had smaller size (about 5 nm) than Zn nanoparticles in Zn@NC. This was because the Bi nanoparticles were evenly redistributed in carbon matrices after in situ replacement of Zn at high pressure during hydrothermal reaction. Meanwhile, lattice spacings of 0.237 and 0.328 nm are assigned to the (104) and (012) planes of the Bi phase, respectively [59]. EDS detection (Fig. S3a) shows that Bi@NC contained the elements Bi, C, N, and O while EDS mapping analysis (Fig. S3b) certifies that Bi, C, and N are homogeneously distributed in Bi@NC. These analyses indicate that the nitrogen atoms in Zn@NC or Bi@NC are derived from ZIF-8. In addition, as depicted in Fig. S2b, the Raman spectrum of Bi@NC exhibited a lower *I*_D_/*I*_G_ ratio (*I*_D_/*I*_G_ = 1.06) than Zn@NC, suggesting a higher degree of graphitization. The dispersion of Bi nanoparticles after replacement led to the reduction of defects in carbon during the hydrothermal reaction.

According to previous reports, nitrogen doping in carbon can enhance the electronic conductivity of carbon matrices and create abundant defects (for instance, nano-pores) on carbon [60, 61]. XPS was performed to determine the nitrogen species in Bi@NC. As shown in Fig. S3c, the N atomic ratio in Bi@NC is about 27.48%, in agreement with EDS analysis. The nitrogen species consisted of pyridinic-*N* (N1, 398.50 eV), pyrrolic-*N* (N2, 399.70 eV), graphitic-*N* (N3, 400.56 eV), and oxidized-*N* (N4, 401.8 eV) [62]. Both pyridinic-*N* and pyrrolic-*N* in NC provide more active sites for lithium ion storage, benefiting mass transport and electron transfer [63].

Figure S3d presents N_2_ adsorption–desorption and corresponding pore diameter distribution curves of Bi@NC. The N_2_ adsorption–desorption isotherm of Bi@NC could be classified as a typical IV (H_3_) isotherm with a distinct hysteresis loop, indicative of the presence of distinct mesoporous microstructures [46]. From the BET result, the specific surface area is 492.08 m^2^ g^−1^ while the single point adsorption total pore volume is 0.2749 cm g^−1^ (*P*/*P*_0_ = 0.9889). According to the narrow pore size distribution in the range 2.1–5 nm, Bi@NC had an average pore size of about 2.23 nm, which was calculated via desorption data using the Barrett–Joyner–Halenda (BJH) model. Obviously, the as-prepared Bi@NC exhibits a porous structure, which is related to its precursor ZIF-8. This porous structure was helpful for volume buffering during lithium insertion/extraction in bismuth. The nanoparticles of bismuth in Bi@NC reduce the distance for lithium transportation in bismuth while the NC increases the electronic conductivity and activity of lithium insertion/extraction. Therefore, the hierarchical configuration of Bi@NC contributes to its excellent electrochemical performances as anode in the lithium ion battery in terms of cyclic stability and rate capability.

**Fig. 6:**
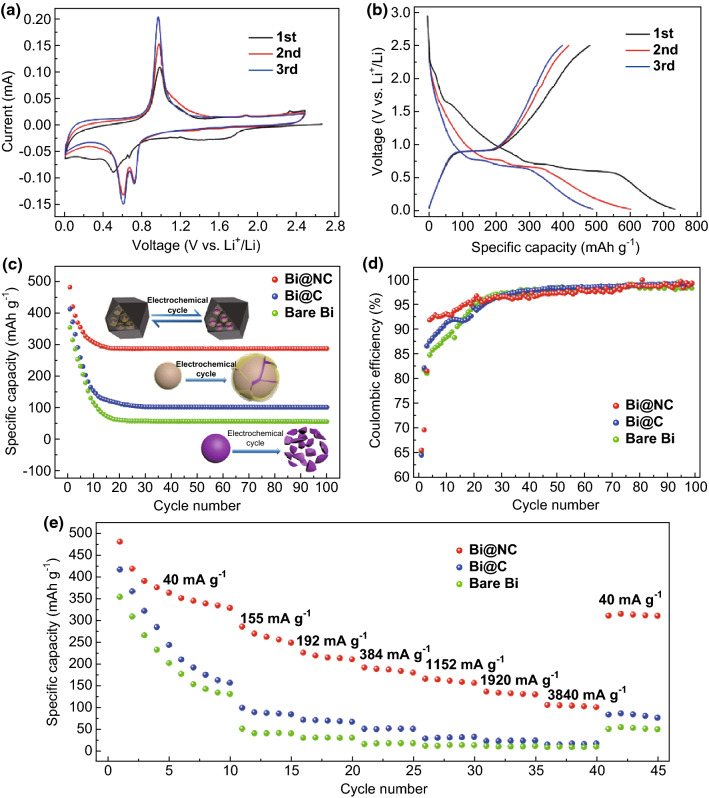
**a** Cyclic voltammograms and **b** charge–discharge curves of Bi@NC; comparisons of **c** cyclic stability, **d** coulombic efficiency, and **e** rate capabilities of Bi@NC, Bi@C, and bare Bi

The cyclic stability and rate capability of the as-prepared Bi@NC were evaluated in a coin cell with metallic lithium as counter electrode. Figure 6a presents the cyclic voltammograms of Bi@NC. Owing to the formation of a solid electrolyte interphase (SEI) layer on the carbon matrix, an irreversible broad peak appeared between 0.01 and 1.8 V during the first cathodic scan [46, 64]. The high specific surface area of Bi@NC and reductive decomposition of the electrolyte led to large irreversible capacity loss during the first cycle [15, 65]. The other two reduction peaks are located at about 0.75 and 0.60 V, which are attributed to the formation of LiBi and Li_3_Bi, respectively [1, 9, 66]. During the anodic process, a sharp peak locates at about 0.9 V is assigned to the reversible extraction of Li^+^ from Li_3_Bi that returned to metallic Bi [18, 22, 67]. These reactions were also identified from charge/discharge tests. Figure 6b presents the charge–discharge curves of the first three cycles of Bi@NC. There are two voltage plateaus at about 0.75 and 0.6 V in the discharge curves, corresponding to the formation of LiBi and Li_3_Bi, respectively. The voltage plateaus around 0.9 V in the charge curves can be ascribed to reversible lithium extraction from Li_3_Bi to Bi. The capacity at a voltage lower than 0.6 V can be ascribed to capacitive contribution from the porous carbon matrix [46].

Figure 6c presents the cyclic stability of Bi@NC at 80 mA g^−1^ after the initial three cycles at 40 mA g^−1^, with comparisons to bare Bi and Bi@C. A drop-off trend of capacity in the initial cycles was distinctly observed in all samples. This is because Bi particles pulverized upon cycling, resulting in the loss of electrical integrity leading to rapid capacity fading [1]. Besides, the sizes of Bi particles in bare Bi and Bi@C (OD = 100 nm) are larger than that in Bi@NC (OD = 5 nm), leading to easier pulverization and faster fading of capacity [21]. The initial first cycle coulombic efficiencies of these three samples (Fig. 6d) are only about 65%, which was ascribed to the formation of SEI on the fresh sample surface during the first cycle. It can be found from Fig. 6c that bare Bi exhibits poor cyclic stability. Its charge capacity decayed quickly before the initial 20 cycles and retained only 60 mAh g^−1^ after 100 cycles. This poor cyclic stability resulted from pulverization of Bi nanoparticles due to their volume change during lithium insertion/extraction and electronic insulation of pulverized particles due to surface SEI [1, 17]. The pulverization of Bi nanoparticles can be clearly indicated by SEM and TEM images of cycled bare Bi, as shown in Fig. S4a. The poor cyclic stability of bare Bi is improved to some extent by coating carbon on Bi nanoparticles. As shown in Fig. 6c, the charge capacity of Bi@C is retained at 100 mAh g^−1^ after 100 cycles. However, this capacity is lower than the theoretical specific capacity of bismuth. Obviously, simple carbon coating did not improve the cyclic stability of bismuth. The large volume change of the bismuth could destroy the carbon-coating layer and expose bismuth to the electrolyte, resulting in pulverization and continuous growth of SEI layers on Bi particle surfaces [68]. The TEM and SEM images in Fig. S4b confirm the destruction of Bi@C particles. Bi@NC, in contrast, showed excellent cyclic stability, with charge capacity (285 mAh g^−1^) that is higher than those of bare Bi or Bi@C. Bi@NC showed excellent cyclic stability with charge capacity (285 mAh g^−1^) that is significantly higher than those of bare Bi or Bi@C. The electrochemical performances of Bi@NC were compared with previous reports in the literature, as displayed in Table S1. The corresponding volumetric capacity is about 430 mAh cm^−3^ (specific capacity × apparent density = 285 mAh g^−1^ × 1.51 g cm^−3^) at current density 80 mA g^−1^, which is also 1.5 times that of graphite (275 mAh cm^−3^, specific capacity × apparent density = 372 mAh g^−1^ × 0.74 g cm^−3^). This excellent performance is attributed to the porous structure of the carbon matrix, which provides space to alleviate the mechanical strain of Bi nanoparticles during lithium insertion/extraction and maintains the structural integrity of Bi (Fig.S5b) [46]. The NC matrix is just like a huge conductive network where the smaller and higher active Bi nanoparticles are anchored, resulting in preferable electrochemical performance compared to bare Bi and Bi@C. Even after 100 cycles, Bi@NC maintains its pristine morphology, as indicated by the TEM and SEM of cycled Bi@NC (Fig. S4c).

Bi@NC exhibited excellent rate capability. Figure 6e presents the rate capability of Bi@NC compared to bare Bi and Bi@C. Obviously, bare Bi and Bi@C almost lost their charge capacities, but Bi@NC delivered a capacity as high as 100 mAh g^−1^ under a high rate current of 3840 mA g^−1^. This excellent rate capability is related to the smaller bismuth nanoparticles uniformly anchored in the NC than those in bare Bi and Bi@C. The smaller nanoparticles shortened the path for lithium transport in the particles and the nitrogen-doped carbon enhanced the electronic conductivity of Bi. It should be noted from Fig. 6c that at low rate current, Bi@NC delivered a charge capacity (over 400 mAh g^−1^) higher than the theoretical specific capacities of bismuth and carbon. This could be ascribed to the capacitive contribution of the high specific surface of the carbon matrix in Bi@NC.

To understand the electrochemical behavior of NC during cycling, its cycle and rate performance were investigated, as shown in Fig. S6a, b. According to previous literature, we propose that Li ions were stored in NC because the Li ions had strong interactions with N atoms [69, 70]. Figure S6a presents the cyclic stability of NC at 80 mA g^−1^ after the initial three cycles at 40 mA g^−1^. The first cycle coulombic efficiency of NC is also low, about 58%. The low coulombic efficiency is attributed to the formation of SEI and storage of Li ions in nanoporous voids, which are difficult to extract [71]. As the cycling at 80 mA g^−1^ proceeded further, the capacity of NC quickly stabilized to exhibited good electrochemical performance with high reversible capacity of about 215 mAh g^−1^ up to 100 charge/discharge cycles. The rate performance of NC was evaluated at various current densities from 80 to 3840 mA g^−1^, as shown in Fig. S6b. As can be seen, the reversible capacities remain stable and decreased regularly with increase in rate. Therefore, there is reason to believe that NC is an excellent carbon matrix that could improve the electrochemical performance of Bi particles in Bi@NC relative to bare Bi or its simple composite with carbon.

**Fig. 7:**
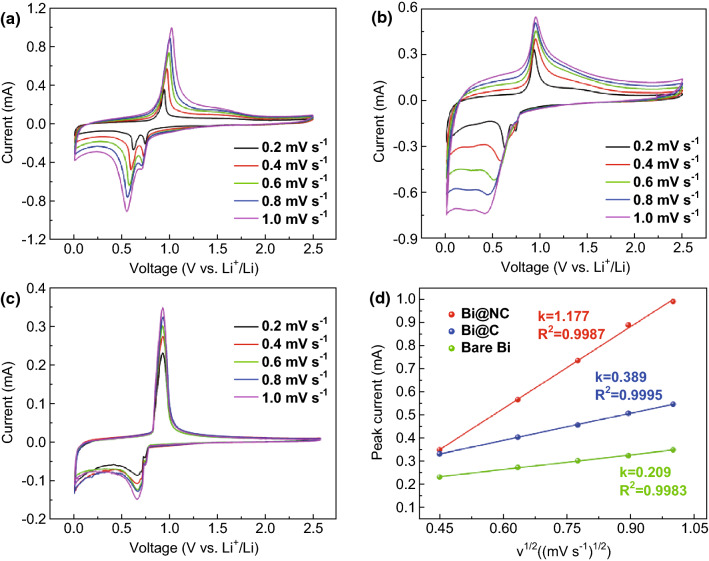
CV characteristics of **a** Bi@NC, **b** Bi@C and **c** bare Biat scanning rates ranging from 0.2 to 1.0 mV s^−1^. **d** Linear relations of anodic peak currents (*i*_p_) versus the square roots of scanning rate (*υ*)

To further understand the kinetic processes of Bi@NC, Bi@C, and bare Bi during lithium insertion/extraction, the lithium ion diffusion coefficient (*D*) was expected. CV characteristics of Bi@NC, Bi@C, and bare Bi at different scanning rates were measured after activation, as shown in Fig. 7a–c. The linear relationship between anodic peak current (*i*_p_) and square root of scanning rate (*υ*) is seen in Fig. 7d. The *D* is extracted by the Randles–Sevcik equation [72]:

$$i_{\text{p}} = 2.69 \times 10^{5} n^{3/2} AD^{1/2} C\upsilon^{1/2}$$

where *i*_p_ refers to the peak current, *n* is the number of electrons in the reaction, *A* is the electrode area, *C* is the concentration of lithium ion in the electrolyte, and *υ* is the scanning rate. The slopes of the fitted lines in Fig. 7d represent the lithium diffusion coefficients. The *D* for Bi@NC cycling from 0.01 to 2.5 V was 7.45 × 10^−7^ cm^2^ s^−1^, which is about 3.0 and 5.5 times larger than those of Bi@C (2.46 × 10^−7^ cm^2^ s^−1^) and bare Bi (1.32 × 10^−7^ cm^2^ s^−1^). These results indicate faster insertion and extraction rate of lithium ions in Bi@NC than in Bi@C and bare Bi, and match the excellent rate performance of Bi@NC. It also proved that NC could increase the insertion and extraction rates of lithium ions in Bi due to the former’s porous structure and nitrogen-doping [64, 73].

The electrochemical impedance test was also measured to examine the kinetic process. In Fig. S7a–c, the electrochemical impendence spectra of Bi@NC, bare Bi, and Bi@C half-cells are presented. The semicircle’s diameter stands for charge-transfer resistance. Although the Bi@NC half-cells had larger internal resistance than the other two in the initial stage, its rate of increase in resistance is slower, which can be clearly observed in Fig. S7d. This is attributed to the differences in the structures of Bi@NC, bare Bi, and Bi@C. In Bi@NC, the Bi nanoparticles are uniformly dispersed in the carbon matrix and most maintain their structural integrities with few SEI layers on the surface after cycling (Fig. S5a, b). In bare Bi and Bi@C, the pulverization of Bi nanoparticles and continuous growth of SEI layers on Bi particle surfaces result in fast growth of resistance.

**Fig. 8:**
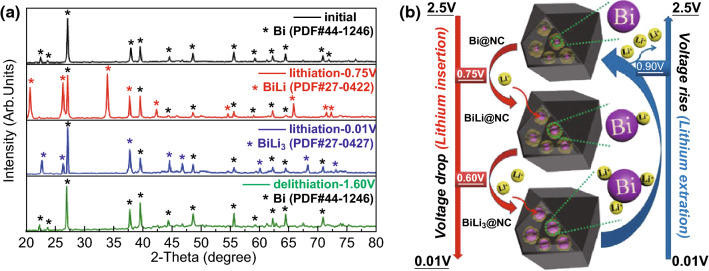
XRD patterns revealing **a** structural and chemical evolution and **b** corresponding mechanism of the Bi@NC electrode

The structural and compositional integrity of Bi@NC was confirmed by identifying its XRD patterns during lithium insertion/extraction. As shown in Fig. 8a, which was obtained during the first charge/discharge process, some Bi is transformed to an LiBi phase when the voltage decreased from 2 to 0.75 V. Li_3_Bi was formed when the voltage was 0.60 V. These reactions were reversible. With these reversible reactions, shown in Fig. 8b, Bi@NC exhibited excellent cyclic stability.

## Conclusions

A novel bismuth–carbon composite, in which bismuth nanoparticles were anchored in nitrogen-doped porous carbon matrices (Bi@NC), was successfully fabricated by galvanic replacement reaction in an MOF (ZIF-8). In this composite, the carbon matrices maintain the morphology of ZIF-8 and exhibit a porous structure, providing space to alleviate the mechanical strain of Bi nanoparticles during Li insertion/extraction. Nitrogen-doped carbon increased the electronic conductivity of the matrix and the reaction activity of bismuth for lithium insertion/extraction. Bismuth nanoparticles uniformly distributed in the carbon matrix reduced the path for lithium transport in the particles. With these features, the as-prepared Bi@NC exhibits excellent cyclic stability and rate capability. The strategy developed in this work solves the cyclic instability issue of bismuth as anode for the lithium ion battery and provides a new approach to high volumetric energy density for electrochemical energy storage devices.

## Electronic Supplementary Material

Below is the link to the electronic supplementary material.

Supplementary material 1 (PDF 1167 kb)
