# Supplementary material for: Bi Nanoparticles Anchored in N-Doped Porous Carbon as Anode of High Energy Density Lithium Ion Battery
Source: Nanomicro Lett. 2018 Jun 8;10(4):56. doi: 10.1007/s40820-018-0209-1 (PMC6199101; doi:10.1007/s40820-018-0209-1)
Supplement: Supplementary file 1 — Supplementary material 1 (PDF 1167 kb) [file 40820_2018_209_MOESM1_ESM.pdf]

Supporting Information for

Bi Nanoparticles Anchored in N-Doped Porous Carbon as Anode of High Energy Density Lithium Ion Battery

Yaotang Zhong[1], Bin Li[1], Shumin Li[1], Shuyuan Xu[1], Zhenghui Pan[1], Qiming Huang[1,2], Lidan Xing[1, 2], Chunsheng Wang[3,]* and Weishan Li[1, 2,]*

[1]School of Chemistry and Environment, South China Normal University, Guangzhou 510006, People's Republic of China

[2]Engineering Research Center of MTEES (Ministry of Education), Research Center of BMET (Guangdong Province), Engineering Lab. of OFMHEB (Guangdong Province), Key Lab. of ETESPG (GHEI), and Innovative Platform for ITBMD (Guangzhou Municipality), South China Normal University, Guangzhou 510006, People's Republic of China

[3]Department of Chemical and Bimolecular Engineering, University of Maryland, College Park, College Park, MD 20740, USA

*Corresponding author. E-mail: cswang@umd.edu (C.S. Wang); liwsh@scnu.edu.cn (W.S. Li)

Supplementary Figures and Table

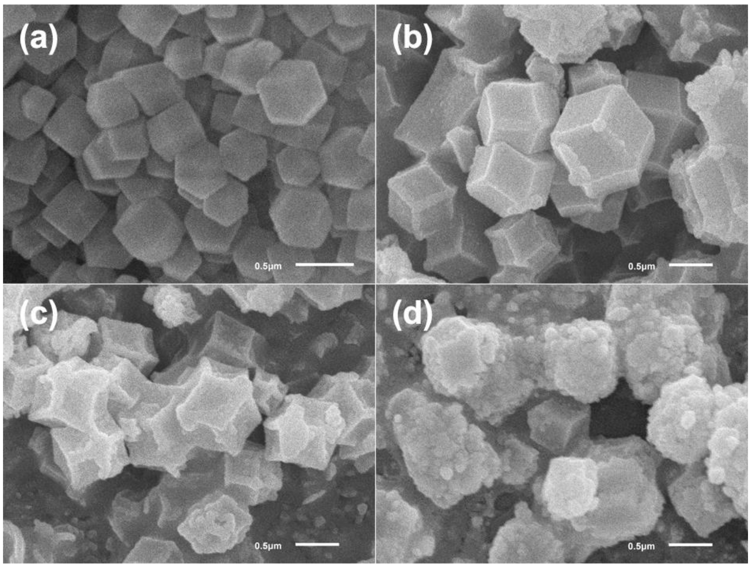

Fig. S1 SEM images of Zn@NC after ZIF-8 calcination at **a** 500 °C, **b** 600 °C, **c** 700 °C and **d** 800 °C

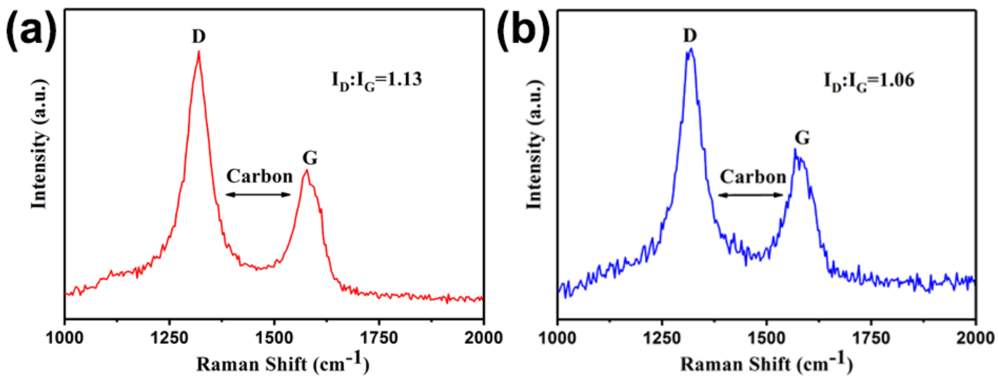

Fig. S2 Raman spectra of **a** Zn@NC and **b** Bi@NC

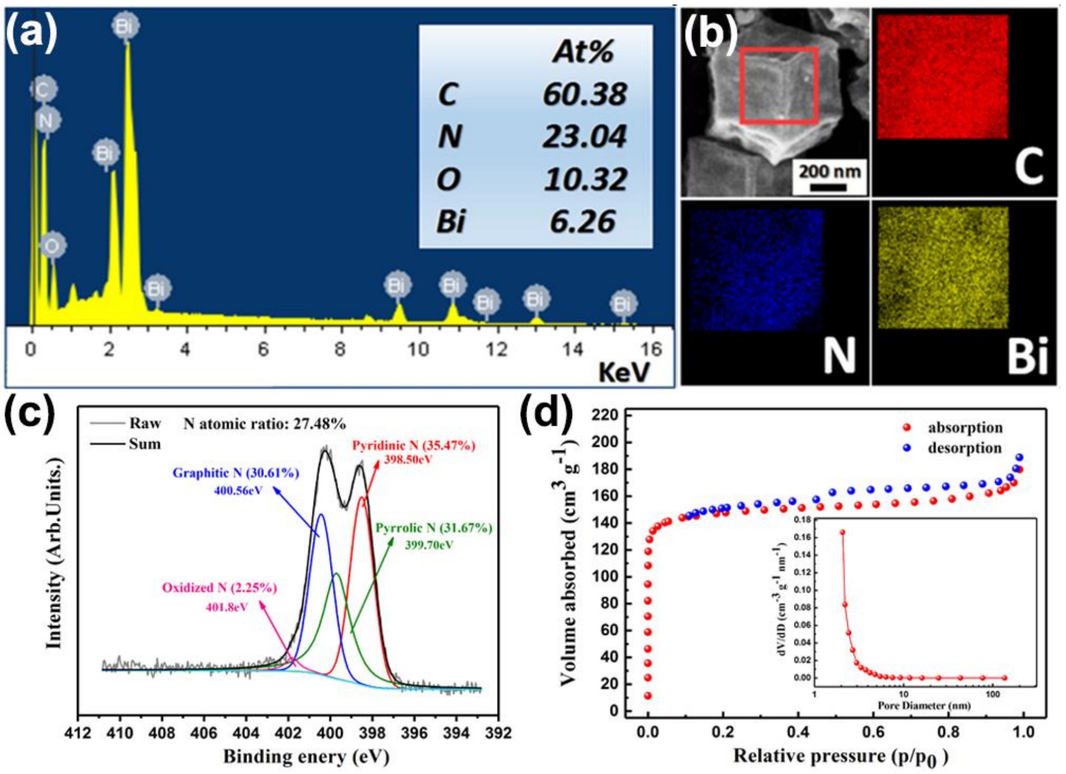

Fig. S3 a EDS pattern and **b** mappings; **c** N 1s XPS spectra, **d** N_2 adsorption-desorption and corresponding pore size distribution curves of Bi@NC

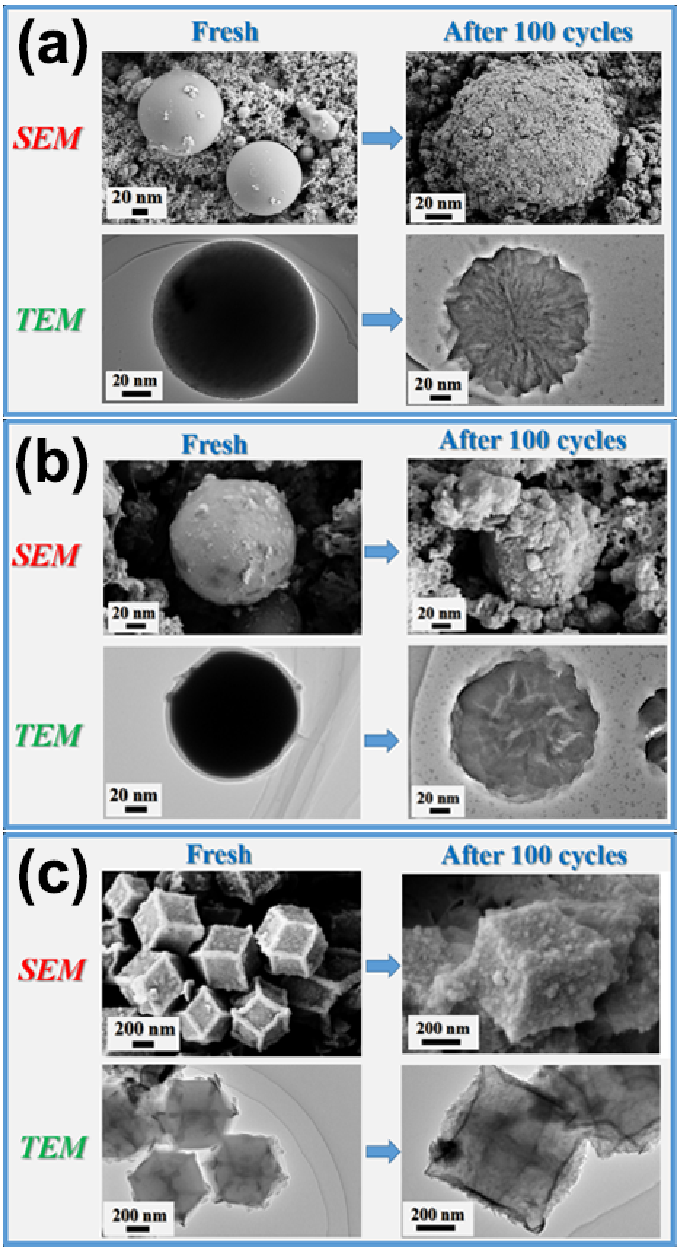

Fig. S4 SEM and TEM images of **a** bare Bi, **b** Bi@C and **c** Bi@NC before and after cycling

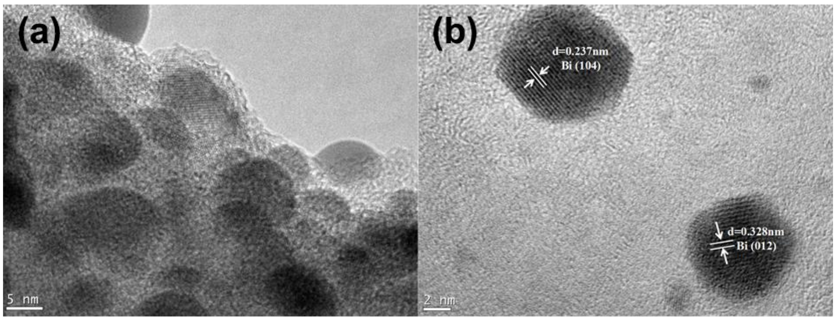

Fig. S5 TEM images of **a** Bi@NC surface and **b** interior after 100 cycles

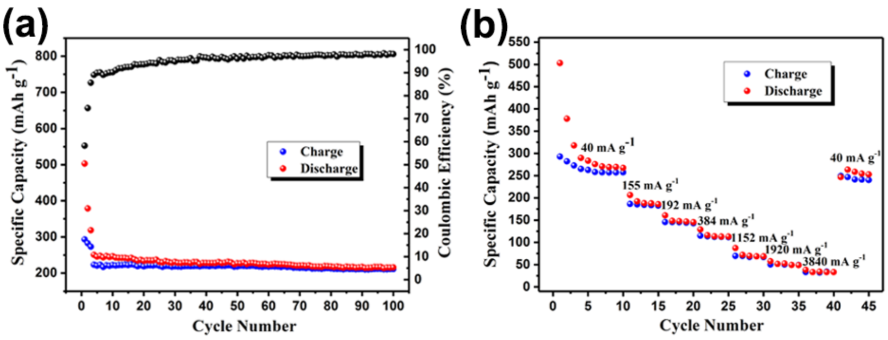

Fig. S6 a Cyclic stability and **b** rate capability of NC

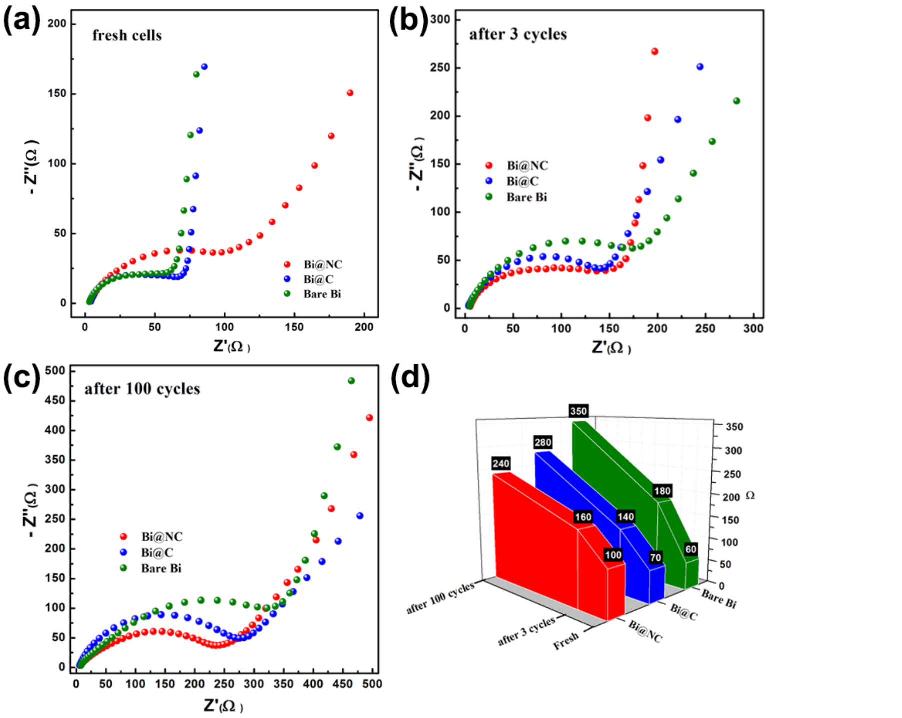

Fig. S7 a, b, c Evolution of electrochemical impedance spectra and **d** interfacial resistances of Bi@NC, Bi@C and bare Bi electrodes before and after cycling

Table S1 Comparisons of electrochemical performance of various electrodes

Electrode	Potential range (V)	Current density (mA g^{-1})	Capacity (mAh g^{-1})	First cycle coulombic efficiency (%)	Reference
Bi@C core-shell nanowires	0.00 ~ 2.5	100	408	63	[15]
Bi@C microspheres	0.01~2.0	100	280	37	[23]
Bi@C nanocomposite	0.00 ~ 2.0	100	300	71	[21]
Bi/Al_2O_3/C	0.00 ~ 2.0	100	310	71	[21]
Bi@NC	0.01~2.5	80	285	65	This work
